# Necrotising fasciitis following abdominoplasty performed abroad: a case report

**DOI:** 10.1016/j.jpra.2026.10.012

**Published:** 2026-10-21

**Authors:** A. Al Sulaiman, J. Alexandre, O. Hermeziu, G. Roccaro, R. Bosc, R. Goron

**Affiliations:** Service de Chirurgie Plastique, Reconstructrice et Esthétique et Maxillo-Faciale, Hôpital Universitaire Henri Mondor, AP-HP, 1 rue Gustave Eiffel, Créteil, 94000, France

**Keywords:** Medical tourism, Necrotising fasciitis, Abdominoplasty, Complication, Case report

## Abstract

**Background:**

Cosmetic medical tourism has grown considerably in Europe, with many patients travelling abroad seeking lower-cost procedures. However, heterogeneous practices and the absence of postoperative follow-up inherent to geographical distance may lead to life-threatening complications with delayed management. We report a case of necrotising fasciitis following abdominoplasty performed abroad.

**Case presentation:**

A 42-year-old female patient underwent abdominoplasty, liposuction, and gluteal lipofilling in a foreign country. The postoperative course was complicated by anaemia, wound dehiscence, and an inflammatory syndrome, initially managed conservatively. Her condition deteriorated, necessitating medical repatriation. On admission, clinical examination revealed abdominal soft tissue necrosis with fever and purulent discharge, alongside a severe inflammatory response on laboratory investigations. Emergency surgical exploration confirmed extensive necrosis of the abdominal fascia and a gluteal collection. Wide debridement with bacteriological sampling and drainage were performed. Empirical broad-spectrum antibiotic therapy was initiated, and postoperative vasopressor support was required. An early second-look procedure was performed the following day. Following vacuum-assisted closure (VAC) therapy, reconstruction with a split-thickness skin graft was carried out. The patient was discharged after six weeks of hospitalisation with significant aesthetic sequelae.

**Clinical relevance:**

Necrotising fasciitis is a rare but potentially fatal condition whose prognosis depends on the promptness of management. Medical tourism, owing to heterogeneous practices and lack of postoperative follow-up, may lead to diagnostic and therapeutic delay, exposing patients to this type of complication. Even in cases of favourable outcome, functional and aesthetic sequelae can be significant.

## Introduction and importance

Cosmetic surgery has expanded markedly over the past three decades, driven by evolving aesthetic standards, improved access to surgical techniques, and the influence of social media. Alongside this trend, cosmetic medical tourism has grown into a widespread phenomenon across Europe and beyond, with patients crossing borders in search of lower costs and shorter waiting times.^1^ Western European countries — including France, the United Kingdom, and Germany — are among the largest sources of outbound cosmetic tourists, with Turkey and Tunisia consistently ranking as preferred destinations.^2^ Packages combining travel, accommodation, and surgery are advertised at prices well below domestic rates, making them attractive to a broad population.^3^

Although precise Europe-wide figures remain difficult to establish, the scale of the phenomenon is significant: several thousand French nationals alone are estimated to seek cosmetic procedures abroad each year,^4^ and comparable patterns have been documented across other Western European countries, most notably in the United Kingdom.^5^ Heterogeneous clinical practices and the absence of adequate postoperative follow-up inherent to geographical distance can result in severe complications, including life-threatening infections, with diagnostic and therapeutic delay upon return home.

Necrotising fasciitis is a rare (0,4/100000 persons per year in Europe/USA) but potentially fatal soft-tissue infection, carrying a mortality of 20–30% even with appropriate multidisciplinary management.^6^ As a complication of aesthetic surgery it remains uncommon: a systematic review of the literature identified only 18 reported cases over three decades, most following liposuction, predominantly monomicrobial and caused by *Streptococcus pyogenes*, with early clinical suspicion and prompt combined antibiotic and surgical management associated with improved survival.^6^ Recognised risk factors include obesity, diabetes, and other causes of impaired immune or vascular status, which may compound the diagnostic and therapeutic delays inherent to treatment abroad. Polymicrobial presentations, although less common, have also been described in the context of cosmetic tourism.^7^

We report a case of life-threatening necrotising fasciitis following abdominoplasty performed abroad in a French patient, illustrating the risks associated with cosmetic medical tourism and the challenges of managing its complications upon repatriation. This case report was prepared in accordance with the SCARE guidelines.

## Case presentation

A 42-year-old female patient with a body mass index (BMI) of 32.7 kg/m² and a history of nephrotic syndrome, a non-smoker on no regular medication, underwent abdominoplasty, liposuction, and gluteal lipofilling on a day-case basis in Tunisia. On postoperative day 3, she developed fever (38 °C) and laboratory evidence of inflammation (white blood cells (WBC) 15,200/mm³, C-reactive protein (CRP) 48 mg/L), for which empirical oral dual antibiotic therapy (ciprofloxacin and metronidazole) was initiated. From day 5, she developed progressive wound dehiscence and livedo-like skin discolouration. Abdominal pain, initially relieved by analgesics, became progressively more intense and unbearable by day 7, prompting her to contact her insurance company for medical repatriation; repeated contact with the treating clinic in the interim resulted only in advice to increase analgesia.

One week after surgery, she was medically repatriated to France and transferred directly by ambulance to our tertiary referral centre. On admission, haemodynamic status was stable (blood pressure 139/84 mmHg, heart rate 119 bpm). Clinical examination revealed abdominal soft tissue necrosis with purulent gluteal discharge (Fig. 1), and laboratory investigations confirmed a severe inflammatory response (WBC 37,600/mm³, CRP 384 mg/L, haemoglobin 9.7 g/dL, sodium 133 mmol/L, creatinine 87 µmol/L, normal blood glucose), yielding a LRINEC score of 10. Necrotising fasciitis was suspected and empirical intravenous broad-spectrum antibiotic therapy (piperacillin–tazobactam and clindamycin) was initiated.

**Fig. 1:**
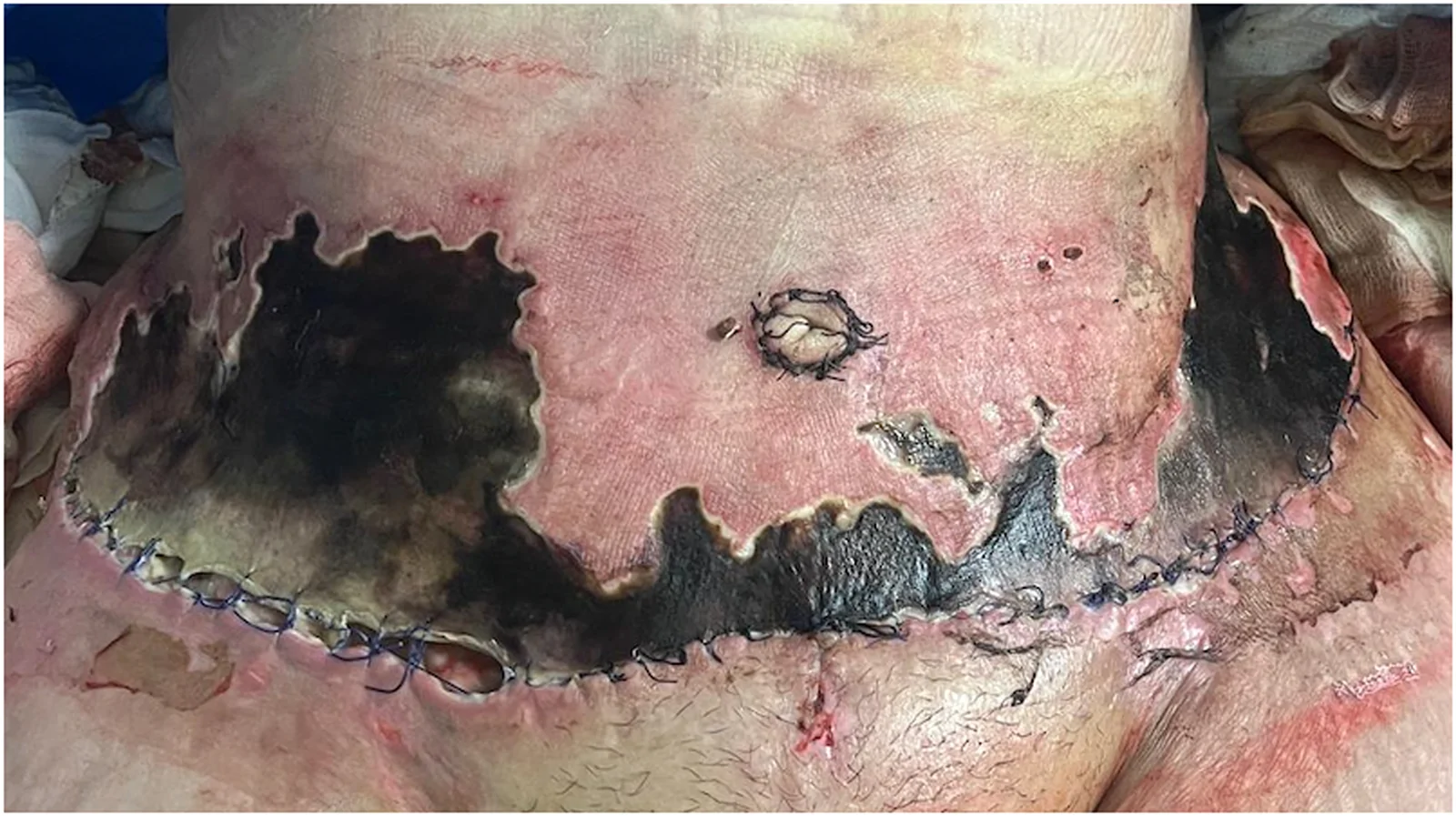
Abdominal necrotic lesions on initial clinical examination.

Emergency surgical exploration confirmed extensive necrosis of the abdominal fascia and a gluteal collection (Fig. 2); the diagnosis of necrotising fasciitis was confirmed macroscopically intraoperatively. Wide debridement, drainage, and bacteriological sampling were performed. Vasopressor support (noradrenaline, up to 2 mg/h) was required for three postoperative days, necessitating admission to the surgical intensive care unit. A second-look procedure the following day allowed further debridement and irrigation, the only further surgical revision required. Cultures identified ESBL-producing *Enterobacter cloacae, Escherichia coli, Enterococcus faecalis*, and *Pseudomonas aeruginosa*, and antibiotic therapy was subsequently switched to imipenem–cilastatin for seven days with a single dose of amikacin. Following transfer to the plastic surgery ward, Vacuum-assisted closure (VAC) therapy was initiated for 29 days, and split-thickness skin grafting was performed immediately after VAC removal, once the wound bed was adequately granulating. The patient was discharged home after several weeks of hospitalisation. She was followed regularly thereafter, at 3 months, 6 months, 1 year, and 2 years postoperatively, and presented with significant aesthetic and psychological sequelae, requiring antidepressant therapy (Fig. 3).

**Fig. 2:**
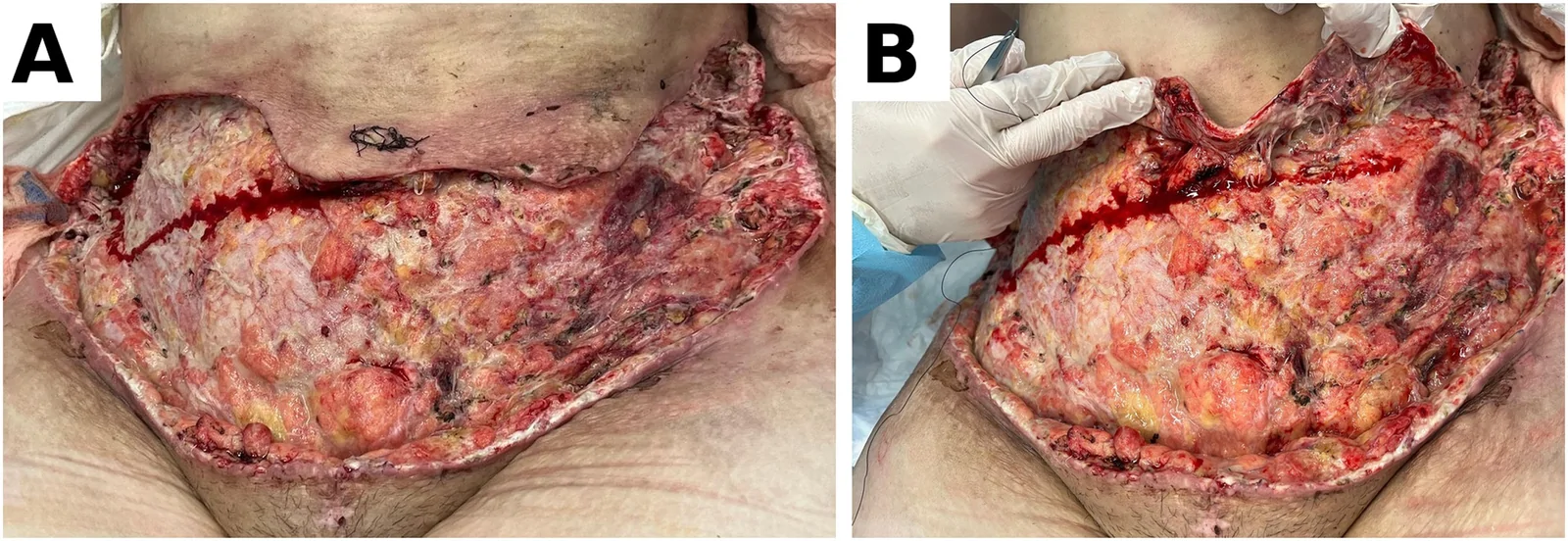
A, B. Intraoperative appearance following wide debridement of necrotic tissue.

**Fig. 3:**
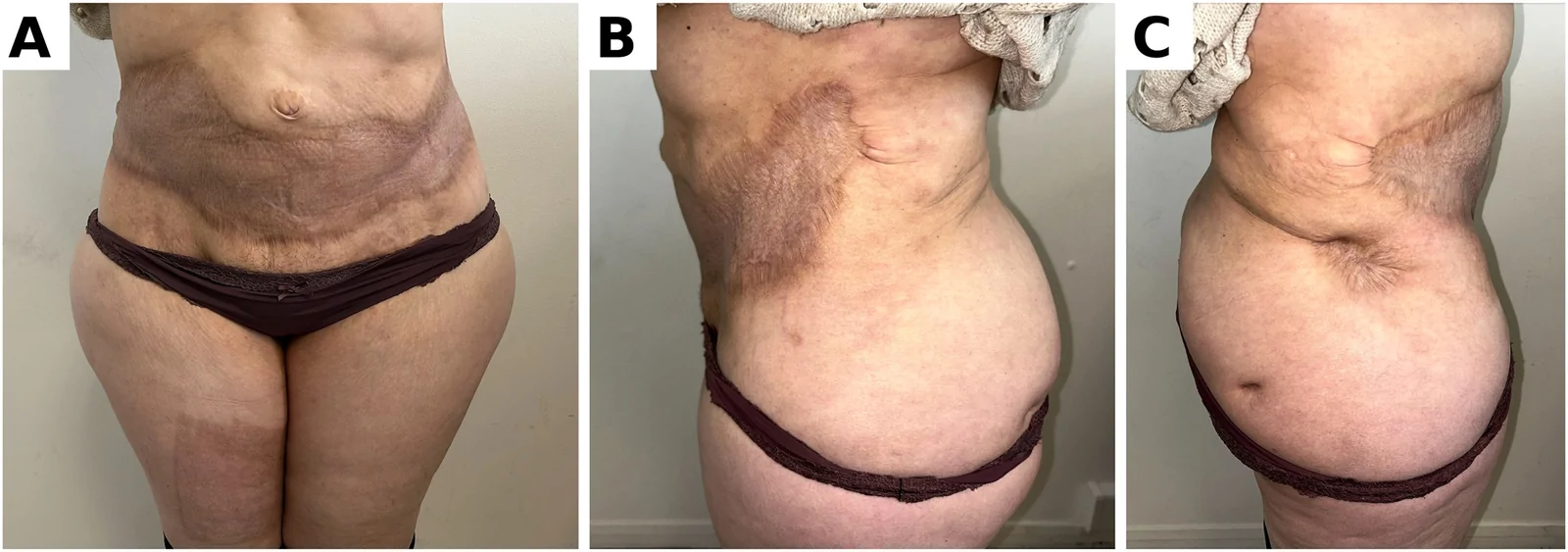
A, B, C. Appearance of the split-thickness skin graft at six months.

## Discussion

This case illustrates the potentially life-threatening consequences of cosmetic surgery performed abroad, with delayed recognition of necrotising fasciitis — a rapidly progressive deep fascia infection carrying a mortality of 20–30% even with appropriate multidisciplinary management.^6^ The context of medical tourism, characterised by early discharge and limited postoperative surveillance, directly contributed to diagnostic delay in this case.

Medical tourism remains a recognised contributing factor in cases such as this. Early discharge and limited postoperative surveillance abroad, followed by delayed access to appropriate care upon return, are consistently associated with more severe presentations and worse outcomes.^8^^,^^9^ Cross-border cosmetic surgery also carries a substantial economic burden for domestic healthcare systems, estimated at several thousand euros per patient in Europe.^10^

Cases of post-cosmetic necrotising fasciitis, though infrequent, have been described in similar contexts of delayed diagnosis and prolonged hospitalisation.^7^ To our knowledge, only few case reports document polymicrobial necrotising fasciitis involving ESBL-producing multidrug-resistant organisms following abdominoplasty performed abroad. The selection of such organisms likely reflects disparities in antibiotic prophylaxis protocols at the operating centre, with additional implications for nosocomial transmission upon repatriation.

Clinicians across Europe must maintain a high index of suspicion for infection in patients returning from surgery abroad. The development of sepsis, livedo-like skin changes, or clinical deterioration despite antibiotic therapy should prompt urgent surgical exploration without delay: timely debridement is the single most important determinant of survival, and delays in managing necrotising fasciitis significantly worsen prognosis.^6^

Early and repeated surgical exploration remains the cornerstone of necrotising fasciitis management: in this case, wide debridement performed at admission was followed by a second-look procedure the following day, allowing rapid control of the infectious process before reconstruction was undertaken. The reconstructive strategy followed a staged approach, with vacuum-assisted closure therapy bridging to split-thickness skin grafting once the wound bed was adequately granulating — an approach well suited to large soft-tissue defects following extensive debridement. At two years’ follow-up, the wound had healed, although significant aesthetic and psychological sequelae persisted, underlining that even a favourable clinical outcome may carry a lasting functional and psychological burden.

This case underscores the importance of early recognition and aggressive multidisciplinary management in necrotising fasciitis, particularly when postoperative surveillance has been limited, as in the context of medical tourism. A high index of suspicion, prompt broad-spectrum antibiotic therapy, and early, repeated surgical debridement remain the key determinants of survival and functional outcome. Clinicians receiving patients after cosmetic surgery performed abroad should maintain a low threshold for surgical exploration when faced with atypical or delayed presentations of severe infection.

## Informed consent

The patient whose case is presented has provided her informed consent for the publication of the manuscript.

## Statement of human and animal rights, or ethical approval

This article does not contain any studies with human participants or animals performed by any of the authors.

## Funding information statement

The authors received no financial support for the research, authorship, and/or publication of this article.

## Declaration of competing interest

None to declare.
